# Reliability of the Dexcom G6 Continuous Glucose Monitor During Hyperbaric Oxygen Exposure

**DOI:** 10.1089/dia.2019.0390

**Published:** 2020-04-27

**Authors:** Enoch Huang, Shaban Demirel, Chanelle Bliss, Davut Savaser, Jessica R. Castle

**Affiliations:** ^1^ Legacy Emanuel Medical Center, Harold Schnitzer Diabetes Health Center, Portland, Oregon.; ^2^ Legacy Research Institute, Harold Schnitzer Diabetes Health Center, Portland, Oregon.; ^3^ Oregon Health and Science University, Harold Schnitzer Diabetes Health Center, Portland, Oregon.

**Keywords:** Hyperbaric oxygen therapy, HBO_2_, Continuous glucose monitors (CGM), Hypoglycemia

## Abstract

***Background:*** People with diabetes-related ulcers may benefit from hyperbaric oxygen (HBO_2_) therapy and from continuous glucose monitors (CGM). Although blood glucose (BG) meters based on glucose oxidase (GO) report erroneously low values at high pO_2_, BG meters based on glucose dehydrogenase (GD) do not. We therefore examined the performance of a GO-based CGM system in comparison to GO-based and GD-based BG systems in normobaric air (NBAir), hyperbaric air (HBAir), and HBO_2_ environments.

***Materials and Methods:*** Twenty-six volunteers without diabetes mellitus (DM) wore Dexcom G6 CGM systems and provided periodic blood samples before, during, and after a standard HBO_2_ treatment consisting of three 30-min intervals of HBO_2_ separated by two 5-min intervals of HBAir. Accuracy of the CGM and GO-based BG meter were assessed by comparisons with the GD-based values.

***Results:*** The mean absolute relative difference for the CGM system was 15.96% and for the GO-based meter was 8.52%. Compared to NBAir, HBO_2_ exposure resulted in significantly higher CGM values (+3.76 mg/dL, *P* < 0.001) and significantly lower GO-based meter values (−10.38 mg/dL, *P* < 0.001). Pre-HBO_2_ and post-HBO_2_ values obtained in NBAir were also significantly different when measured by CGM (+4.13 mg/dL, *P* = 0.015) or the GO-based meter (−9.04 mg/dL, *P* < 0.001).

***Conclusions:*** In volunteers without DM, HBO_2_ exposure results in statistically significant differences in glucose measurements obtained with GO-based devices, but not a GD-based device. Standard HBO_2_ treatment results in statistically significant effects on glucose concentrations. These differences are of unlikely clinical significance.

## Introduction

Diabetes mellitus (DM) is an epidemic of global proportion with a steadily rising prevalence. It is believed that at least 15% of people living with DM will develop a lower extremity foot ulcer during their lifetime.^1,2^ The annual incidence of foot ulcer development, which often requires hyperbaric oxygen (HBO_2_) therapy,^3^ is 5%–6% among people living with DM.^1,2,4^ However, HBO_2_ is thought to lower estimated glucose values (EGVs) over the course of a treatment,^5–15^ leading to treatment protocols that rely on pre-HBO_2_ EGV to determine whether patients can be safely treated that same day or not.^16^ This usually involves multiple finger sticks performed with point-of-care (POC) glucometers. For multiplace hyperbaric facilities, patients may have their blood glucose (BG) tested during therapy; however, this is not a possibility for patients in monoplace hyperbaric facilities.

Continuous glucose monitors (CGM) allow monitoring of EGV without the need for multiple finger sticks.^17,18^ Patients have a sensor inserted transcutaneously with a sensor wire that is located in the interstitial subcutaneous space. This sensor wire uses a glucose oxidase (GO) chemical reaction to generate an electrical signal that is converted into an EGV. A transmitter is attached to the sensor that records the EGV and transmits the data to a receiver or smartphone. The transmitter has a 3-h storage capacity in case there is a temporary disconnection with the receiver. Missing data can be backfilled when the data connection is restored. CGM offers a unique opportunity to monitor EGV before, during, and after HBO_2_, but CGM reliability in a HBO_2_ environment has not been established. To date, there have been only two publications – one in vitro and one in vivo – reporting the effects of hypobaric and hyperbaric chamber exposures on a CGM, however, those studies were done with air and not oxygen.^19,20^

A patient receiving HBO_2_ may have alveolar pO_2_ over 1800 mmHg with tissue pO_2_ over 1000 mmHg. POC glucometers utilizing GO test strips have been shown to underestimate glucose levels when exposed to high pO_2_ levels,^21–24^ while those using glucose dehydrogenase (GD) test strips are not affected by high pO_2_ levels.^24^ Hyperbaric protocols often provide high glycemic index carbohydrates to patients who do not have a minimum pretreatment glucose level.^16^ Underestimation of blood glucose levels (BGLs) may result in unnecessary carbohydrate supplements and iatrogenic hyperglycemia. Before EGVs produced by CGMs that utilize a GO reaction are used in clinical decision making it is imperative that their accuracy be established.

## Materials and Methods

A commercial Dexcom G6 CGM (Dexcom, Inc., San Diego, CA) was placed on individuals without DM who were given a standard HBO_2_ treatment at 2.4 atmospheres absolute (ATA) in a multiplace chamber (OxyHeal Systems, San Diego, CA) using three 30-min oxygen breathing periods separated by two 5-min air breaks. We compared the CGM results against the hospital's Nova StatStrip POC glucometer (Nova Biomedical, Waltham, MA), which uses an ion selective electrode GO reaction, and a Contour Next POC glucometer that uses a flavin adenine dinucleotide (FAD) GD reaction (Ascensia Diabetes Care, Parsipanny, NJ). We obtained concurrent transcutaneous oximetry measurements (TCOM) no farther than 3 cm from the insertion site of the CGM to confirm elevated tissue pO_2_. We calculated differences in EGV between the three different pressure+inhaled gas conditions (normobaric air [NBAir], hyperbaric air [HBAir], and HBO_2_). Linear mixed effects regression models were used to perform the statistical analyses as they can account for repeated measurements made from each participant. Our fitted models allowed for individuals to have different overall levels of estimated glucose.

### Mean absolute relative difference

As GD glucometers are not affected by pO_2_,^24^ we used the Contour glucometer as a reference standard. We calculated the mean absolute relative difference (MARD) of the Dexcom and Novastat glucometer readings against the Contour at each of the time points.

### Blood sampling

We obtained deep fingerstick capillary blood samples for testing using a 28-gauge lancet. When in the hyperbaric chamber, blood samples were drawn up using a micro-pipette in preparation for transfer through a medication transfer lock. Testing of the blood samples was performed immediately upon removal from the medication lock, a process that took less than 2 min from the time the blood was collected.

### Measurements

#### BG readings

All participants agreed to refrain from any oral intake of food or liquids other than water for 2 h before the study period. This was done to ensure that any fluctuations in BG were unrelated to carbohydrate consumption.

**FIG. 1.**
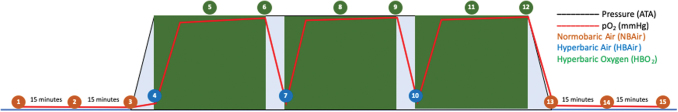
Glucose testing protocol. Fingerstick blood glucose sampling was performed at each of the time points during a standard hyperbaric treatment to 2.4 ATA. Yellow circles correlate to breathing NBAir (pO_2_ = 159.6 mmHg), blue circles correlate with breathing HBAir (pO_2_ = 383.0 mmHg), and green circles correlate with breathing HBO_2_ (pO_2_ = 1824 mmHg). ATA, atmospheres absolute; HBAir, hyperbaric air; NBAir, normobaric air. Color images are available online.

CGM data were recorded at 5-min intervals and wirelessly transmitted to a Dexcom receiver. CGM and POC glucometer readings were recorded at multiple time points (Fig. 1) representing either NBAir (pO_2_ of 159.6 mmHg), HBAir (pO_2_ of 380 mmHg) and HBO_2_ (pO_2_ of 1824 mmHg). The schedule for blood sampling was as follows:

(1) 30 min Pre-HBO_2_ at 1 ATA (NBAir)
(2) 15 min Pre-HBO_2_ at 1 ATA (NBAir)
(3) Immediately Pre-HBO_2_ at 1 ATA (NBAir)
(4) Reaching 2.4 ATA on air (HBAir)
(5) Mid-O_2_ period 1 at 2.4 ATA (15 min of O_2_) (HBO_2_)
(6) Post-O_2_ period 1 at 2.4 ATA (30 min of O_2_) (HBO_2_)
(7) Pre-O_2_ period 2 at 2.4 ATA (HBAir)
(8) Mid-O_2_ period 2 at 2.4 ATA (45 min of O_2_) (HBO_2_)
(9) Post-O_2_ period 2 at 2.4 ATA (60 min of O_2_) (HBO_2_)
(10) Pre-O_2_ period 3 at 2.4 ATA (HBAir)
(11) Mid-O_2_ period 3 at 2.4 ATA (75 min of O_2_) (HBO_2_)
(12) Post-O_2_ period 3 at 2.4 ATA (90 min of O_2_) (HBO_2_)
(13) Immediately Post-HBO_2_ at 1 ATA (NBAir)
(14) 15 min Post-HBO_2_ at 1 ATA (NBAir)
(15) 30 min Post-HBO_2_ at 1 ATA (NBAir)

### Transcutaneous tissue oximetry

We obtained concurrent TCOM using a Clarke electrode (Radiometer America, Brea, CA) attached no farther than 3 cm from the Dexcom CGM to measure tissue oxygenation in the region of the CGM. The TCOM value closest to the time of each blood draw was used for analysis.

### Participant recruitment

To minimize fluctuations in BG as a result of underlying DM, we recruited individuals without DM who were willing to undergo a single hyperbaric exposure to 2.4 ATA while breathing 100% oxygen. Participants provided their consent to be included in the study after being informed of the study procedures and the risks of participation. Upon enrollment, participants had the Dexcom G6 CGM electrode inserted into the skin of their abdomen. They were scheduled to return >48 h after CGM insertion for their hyperbaric exposure. We planned for the recruitment of at least 24 participants.

The inclusion criteria required participants to be willing to participate in all study-related activities. The exclusion criteria included self-reported diagnosis of DM or borderline DM, contraindications to HBO_2_, or contraindications to having a CGM electrode inserted.

## Results

**FIG. 2.**
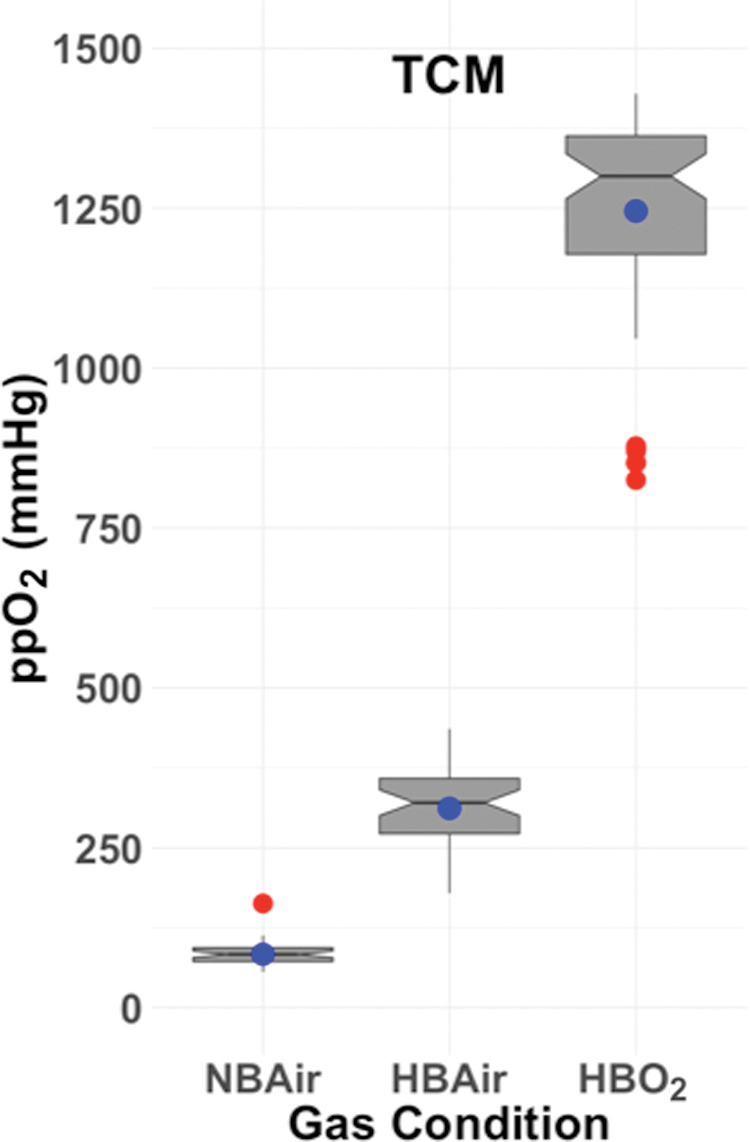
Box plots of TCOM for each gas+pressure condition. There was a significant change in TCOM between NBAir, HBAir, and HBO_2_. The notch displays the 95% confidence interval around the median value, the blue dot shows the mean value, the box covers the IQR (25th percentile to 75th percentile), the whiskers add 1.5 times the IQR to the 75 percentile and subtract 1.5 times the IQR from the 25 percentile, and the red dots show outliers. IQR, interquartile range; TCOM, transcutaneous oximetry measurements. Color images are available online.

We enrolled 26 participants but excluded 3 from analysis due to missing or anomalous data. One participant withdrew without undergoing HBO_2_ exposure, one had missing CGM data during the HBO_2_ window, and one had anomalous TCOM data during the period of HBO_2_ caused by a loose TCOM electrode. Mean TCOM values were significantly different between NBAir, HBAir, and HBO_2_ (Fig. 2). Characteristics of the 23 participants whose data were analyzed are summarized in Table 1.

**Table 1.** Characteristics of Study Participants

Number of participants	26 (3 excluded)
Male	9 (39.1%)
Female	14 (60.9%)
Mean age (years)	40
Median age (years)	37
Range (years)	24–68

**FIG. 3.**
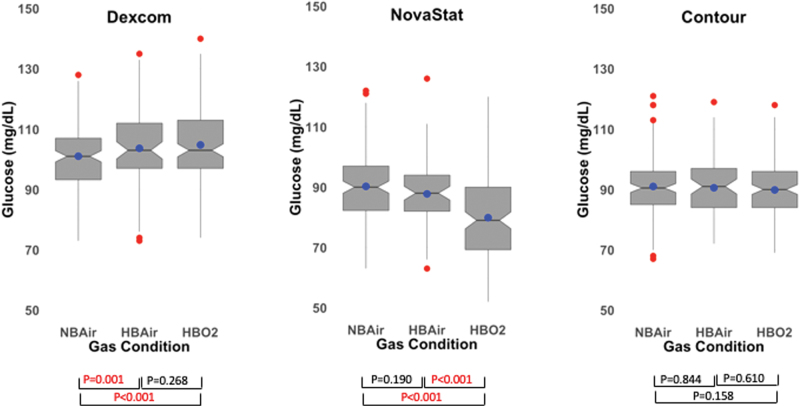
Box plots of EGV for each gas+pressure condition by glucometer. There was a significant change between NBAir and HBO_2_ for the Dexcom and NovaStat, but not the Contour. The notch displays the 95% confidence interval around the median value, the blue dot shows the mean value, the box covers the IQR (25th percentile to 75th percentile), the whiskers add 1.5 times the IQR to the 75 percentile and subtract 1.5 times the IQR from the 25 percentile, and the red dots show outliers. EGV, estimated glucose value. Color images are available online.

EGVs for the Dexcom CGM, the Novastat, and the Contour glucometers are listed in Table 2 and displayed in Figure 3.^25,26^ When comparing HBAir versus NBAir, the Dexcom showed a rise of 2.64 mg/dL (2.61%, *P* = 0.002), the Novastat showed a drop of 2.51 mg/dL (2.79%, *P* = 0.141), and the Contour had a drop of 0.45 mg/dL (0.49%, *P* = 0.841). When comparing HBO_2_ versus HBAir, the Dexcom showed a rise of 1.12 mg/dL (1.08%, *P* = 0.320), the Novastat showed a drop of 7.86 mg/dL (8.96%, *P* < 0.001), and the Contour had a drop of 0.72 mg/dL (0.79%, *P* = 0.644). When comparing HBO_2_ versus NBAir, the Dexcom showed a rise of 3.72 mg/dL (3.72%, *P* < 0.001), the Novastat showed a drop of 10.38 mg/dL (11.50%, *P* < 0.001), and the Contour had a drop of 1.17 mg/dL (1.28%, *P* = 0.177).

**Table 2.** Estimated Glucose Values for Each Glucometer at Each Pressure/Gas Combination from a Linear Mixed Effects Regression Model

	NBAir (mg/dL)	HBAir (mg/dL)	HBO_2_ (mg/dL)	HBAir – NBAir (mg/dL)	% Change	P	HBO_2_-HBAir (mg/dL)	% Change	P	HBO_2_-NBAir (mg/dL)	% Change	P
Dexcom	101.03	103.70	104.79	2.64	2.61%	0.002	1.12	1.08%	0.320	3.72	3.72%	<0.001
Novastat	90.27	87.75	79.89	−2.51	−2.79%	0.141	−7.86	−8.96%	<0.001	−10.38	−11.50%	<0.001
Contour	91.01	90.57	89.85	−0.45	−0.49%	0.841	−0.72	−0.79%	0.644	−1.17	−1.28%	0.177

HBO_2_, hyperbaric oxygen; HBAir, hyperbaric air; NBAir, normobaric air.

EGVs for the Dexcom CGM, Novastat, and Contour glucometers immediately before and after HBO_2_ are listed in Table 3. The Dexcom CGM showed a rise of 4.13 mg/dL (4.13%, *P* = 0.015), the Novastat showed a drop of 9.04 mg/dL (9.84%, *P* < 0.001), and the Contour showed a drop of 5.00 mg/dL (5.43%, *P* = 0.039).

**Table 3.** Estimated Glucose Values for Each Glucometer Immediately Before and Immediately After HBO_2_ from a Linear Mixed Effects Regression Model

Glucometer	Before HBO_2_ (mg/dL)	After HBO_2_ (mg/dL)	Difference	%	P
Dexcom CGM	99.91	104.04	4.13	4.13	0.015
Novastat	91.87	82.83	−9.04	−9.84	<0.001
Contour	92.09	87.09	−5.00	−5.43	0.039

CGM, continuous glucose monitoring.

When using the Contour as a reference, the cohort average MARD for the Dexcom was 15.96% (standard deviation (SD) 10.96%, Range 4.50% to 45.50%), whereas the cohort average MARD for the Novastat was 8.52% (SD 2.39%, Range 4.19% to 13.27%).

## Discussion

The effects of HBO_2_ on EGV in patients with DM has been described in multiple publications. Springer first reported a mean drop of 51 mg/dL in 25 patients with DM after HBO_2_.^13^ Capelli-Schellpfeffer et al. reported a decrease of 21 mg/dL in seven patients with DM.^12^ Al-Waili et al. reported that HBO_2_ lowered EGV by 23% in a series of 41 patients with DM and hypertension undergoing HBO_2_.^6^ Ekanayake and Doolette reported that five patients with DM experienced an average drop of 3.5 ± 0.7 mmol/L (63 mg/dL) after HBO_2_.^27^ The first group to conclude that there was no significant change in BG after HBO_2_ was Rose et al., who found a maximum decrease of 17 mg/dL after the first HBO_2_ treatment and a rise of 5 mg/dL after HBO_2_ after the 10th treatment. They did report that the mean pre-HBO_2_ fasting glucose decreased from 219 to 187 mg/dL (a drop of 32 mg/dL) between the first HBO_2_ treatment and the tenth HBO_2_ treatment.^14^ Niezgoda et al. reported an average decline of 62.7 mg/dL in 272 HBO_2_ treatments.^9^ Trytko reported that patients with DM experienced a mean reduction of BGL of ∼2 mmol/L (36 mg/dL) with a single HBO_2_ treatment.^7^ A reduction in BGL was seen in 84% of treatments involving patients with insulin-dependent DM and 77% of treatments involving patients with noninsulin-dependent DM. Fife et al. reported a decrease of between 39 and 47 mg/dL after HBO_2_,^10^ and Perdrizet et al. reported a decrease of between 17 and 37 mg/dL.^11^ Wilkinson showed a statistically significant decrease in glucose after receiving HBO_2_, but did not report a numerical value.^15^ Most recently, Heyboer et al. found a median decrease of 25 mg/dL in 75.4% of patients with DM after receiving HBO_2_, but concluded that HBO_2_ did not cause a significant drop in BG in patients with DM.^8^

Of significance, only three of these studies specified the methodology of their glucose testing. Ekanayake et al. tested venous blood using a hexokinase reaction that is unaffected by ambient oxygen,^27,28^ Rose et al. used a GO-based glucometer that had previously been validated against a Yellow Springs Instruments laboratory glucometer, showing that it was unaffected by high environmental oxygen levels.^21,29^ It is interesting to note that Rose et al. demonstrated the smallest variation in pre- versus post-HBO_2_ glucose measurements including a rise in mean BG after the 10th treatment.^14^ Heyboer disclosed to this author (E.H.) that their study glucometer used a GD reaction (personal communication).

The effect of HBO_2_ on the BG of patients without DM has been reported by only a few investigators. Capelli-Schellpfeffer et al. recorded a drop of only 0.57 mg/dL in 11 patients. Ekanayake and Doolette stated that there was no change in BG, but did not report actual values.^27^ Wilkinson et al. reported no change in glucose in obese men without DM, but did not report a numerical value.^15^ Interestingly, this group utilized a hyperinsulinemic euglycemic clamp to study overweight and obese men both with and without DM who were exposed to a series of HBO_2_ treatments, demonstrating an increase in insulin sensitivity in the entire cohort of patients.^15^ In an earlier study of nonobese men without DM who received a course of 30 HBO_2_ sessions, they reported a statistically significant decrease (no numerical value provided) in Hba1c.^30^

**FIG. 4.**
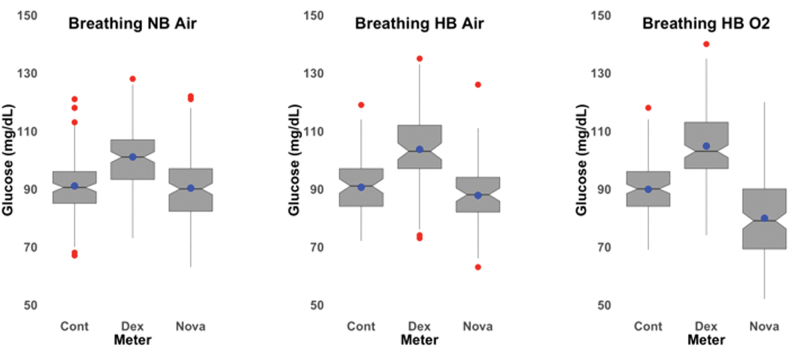
Box plots of EGV for each glucometer by gas+pressure condition. The Dexcom CGM had a positive bias compared to the two POC glucometers at each gas+pressure condition. The notch displays the 95% confidence interval around the median value, the blue dot shows the mean value, the box covers the IQR (25th percentile to 75th percentile), the whiskers add 1.5 times the IQR to the 75 percentile and subtract 1.5 times the IQR from the 25 percentile, and the red dots show outliers. Dex = Dexcom CGM; Nova = Novastat; Cont = Contour. CGM, continuous glucose monitoring; POC, point-of-care. Color images are available online.

Our CGM results revealed a statistically significant increase in EGV when transitioning from NBAir to HBAir and from NBAir to HBO_2_; however, the change in EGV that we observed (+3.72 mg/dL, *P* < 0.001) would generally not be considered clinically relevant (Fig. 3). The Novastat POC glucometer showed a 10.38 mg/dL (11.5%, *P* < 0.001) reduction in EGV when transitioning from NBAir to HBO_2_, which may be clinically relevant if patients are on a HBO_2_ protocol that requires administration of additional carbohydrates before or during treatment. These results confirmed our suspicion that the hospital's GO-based glucometer underestimated glucose values under hyperoxic conditions. This raises concerns about inappropriate carbohydrate supplements being given to patients with DM while they undergo HBO_2_, potentially leading to unnecessary episodes of iatrogenic hyperglycemia. As expected, the Contour glucometer showed practically no effect of pressure or gas combination on EGV (−1.17 mg/dL, −1.28%, *P* = 0.177). The insensitivity of this device to changes in atmospheric or tissue oxygen levels is related to its use of FAD GD, rather than GO, as the catalyst. EGVs from the CGM devices were consistently higher than EGVs from either POC glucometer (Fig. 4), which was unexpected given the low bias of Dexcom G6 values with respect to reference values from venous blood as reported in the labeling of the device.^31^ The higher MARD for the Dexcom reflects the positive bias in EGV readings using the CGM.

The previous in vivo report of hypobaric and hyperbaric exposures on CGM function was on a single research participant without DM who had 48 Medtronic Enlite™ sensors (Medtronic, Inc., Northridge, CA) implanted on his abdomen and back over two successive days while breathing air. The first 24 sensors were tested in a hypobaric environment (0.5 ATA, pO_2_ of 79.8 mmHg) on day 1, while the last 24 sensors were tested in a hyperbaric environment (4 ATA, pO_2_ of 638.4 mmHg) on day 2. The Enlite™ CGM had less accuracy during hypobaric exposures than hyperbaric exposures, and this was theorized to be secondary to microbubbles that developed during sensor insertion. Any bubbles would increase in volume with a decrease in ambient pressure, decreasing the amount of contact—and therefore decreasing accuracy—between the sensor wire and the surrounding interstitial fluid. Conversely, any microbubbles that formed during insertion would have decreased during pressurization to 4 ATA, reducing any issues that may have been present and increasing accuracy.^19^ Similarly, pressurization to 2.4 ATA in our study would have reduced any microbubble volume, and breathing 100% O_2_ would have rapidly eliminated any residual microbubbles over the course of the first 30 min of the treatment.

Our data show that HBO_2_ has a minimal effect on the EGV of individuals without DM, but one that is the opposite of what was predicted. Despite utilizing a GO reaction, the Dexcom G6 CGM does not appear to underestimate glucose values when exposed to high oxygen concentrations. To ensure that glucose is the rate-limiting reactant and that oxygen is present in excess, amperometric CGM sensors based on GO typically surround the working electrode with a glucose-limiting membrane.^32^ The ambient temperature of the sensor wire can also affect EGV, with a 3%–4% increase in EGV with each 1°C increase in temperature (unpublished data). Ambient temperature in the hyperbaric chamber can fluctuate between 20°C and 32.2°C with smaller fluctuations on body temperature. We did not collect sensor temperature to determine the effects of the hyperbaric treatment on body temperature. HBO_2_ can cause peripheral vasoconstriction, increase systemic vascular resistance and blood pressure, and increase the work of breathing because of the increased density of the inspired gas.^33^ It is unclear why these physiologic changes would affect interstitial temperature or glucose and needs to be further investigated. Future studies will need to record interstitial fluid temperature.

### Limitations

One limitation of our study is that we did not compare any of the CGM or POC readings to a laboratory standard. This is especially relevant given the 2013 Clinical and Laboratory Standards Institute (CLSI) POC testing standards that state:

- At <100 mg/dL, 95% of results should be within ±12 mg/dL
- At ≥100 mg/dL, 95% of results should be within ±12.5%
- At ≤75 mg/dL, 98% of results should be within ±15 mg/dL
- At >75 mg/dL, 98% of results should be within ±20%.^24^

This results in a 25%–40% range of “acceptable” error and potentially makes POC glucometers unreliable as a reference standard. This could explain why we had a positive bias in EGV and higher-than-expected MARD for the CGM. Future studies will need to have EGVs compared to a standardized reference laboratory value.

We did not directly measure blood pO_2_, but instead used TCOM as a surrogate marker for tissue hyperoxia. TCOM may actually be more representative of the interstitial environment than venous or arterial pO_2_, but it does not represent arterial or venous pO_2_. Blood pO_2_ drops rapidly when exposed to air, but there was a very minimal surface area in the capillary tubes that was exposed to air, and the blood samples were tested within minutes of removal from the hyperbaric chamber. It is unlikely that the pO_2_ in the blood samples would have dropped significantly between sampling and testing, but this change has not been quantified. Future studies may benefit from blood collection in air-tight syringes to measure blood pO_2_ to verify hyperoxia.

The EGV generated by the GO reaction used by the CGM sensor should be independent of hyperoxia, but we could not independently verify this to be true. This study did not isolate the GO reaction from a physiologic change in EGV related to HBO_2_. Therefore, the changes in EGV could either be explained by an intrinsic error in the glucometers caused by hyperoxia, or it could represent a direct physiologic effect of HBO_2_ on EGV in people without DM. The fact that the two glucometers using a GO reaction detected changes while the GD reaction glucometer detected no change argues against a direct physiologic effect. In vitro testing of the GO reaction using standardized glucose solutions under hyperbaric conditions is warranted.

We did not perform testing of this device in a monoplace hyperbaric chamber where the patient's entire body is in a 100% O_2_ environment because it was not possible to obtain fingerstick BG samples during HBO_2_ exposure. However, exposure to 100% ambient O_2_ should not affect the readings of the CGM as the sensor wire is completely inserted in the subcutaneous space. Although we did not use the CGM in a monoplace hyperbaric chamber, safety evaluation and testing showed that this CGM should not pose a risk of fire if used in a 100% HBO_2_ environment.^34^

We did not test CGMs manufactured by other companies because they had not previously been cleared for hyperbaric use, or they did not have the capacity to transmit data wirelessly through the hull of our multiplace hyperbaric chamber. Because of differences in manufacturing and sampling algorithms, the results from this study should not be extrapolated to other manufacturers' CGMs. Future studies would benefit from comparison to other manufacturers' CGMs.

## Conclusions

In people without DM, there is a statistically significant—but clinically insignificant—effect of gas+pressure condition on the glucose estimate detected by the Dexcom G6 CGM system. Exposure to HBO_2_ was associated with a statistically significant and potentially clinically relevant decrease in glucose estimate reported by the Novastat glucometer. There is a measurable effect of HBO_2_ on the EGV of people without DM; however, it is not clinically significant.
